# Development of a Genetically Encoded Magnetic Platform in Magnetospirillum gryphiswaldense MSR-1 for Downstream Processing of Protein Expression System

**DOI:** 10.21203/rs.3.rs-2630343/v1

**Published:** 2023-03-13

**Authors:** Sha Wu, Jiesheng Tian, Xianle Xue, Zongwen Tang, Zekai Huang, Bruce D. Hammock, Christophe Morisseau, Qing X. Li, Ting Xu

**Affiliations:** China Agricultural University; China Agricultural University; China Agricultural University; China Agricultural University; China Agricultural University; University of California; University of California; University of Hawaii at Manoa; China Agricultural University

**Keywords:** Magnetospirillum gryphiswaldense MSR-1, Magnetosome, Protein purification, Downstream processing, Nanobody

## Abstract

**Background:**

Protein downstream processing remains a challenge in protein production, especially in low yields of products, in spite of ensuring effective disruption of cell and separation of target proteins. It is complicated, expensive and time-consuming. Here, we report a novel nano-bio-purification system for producing recombinant proteins of interest with automatic purification from engineered bacteria.

**Results:**

This system employed a complete genetic engineering downstream processing platform for proteins at low expression levels, referred to as a genetically encoded magnetic platform (GEMP). GEMP consists of four elements as follows. (1) A truncated phage lambda lysis cassette (RRz/Rz1) is controllable for lysis of *Magnetospirillum gryphiswaldense* MSR-1 (host cell). (2) A surface-expressed nuclease (NucA) is to reduce viscosity of homogenate by hydrolyzing long chain nucleic acids. (3) A bacteriogenic magnetic nanoparticle, known as magnetosome, allows an easy separation system in a magnetic field. (4) An intein realizes abscission of products (nanobodies against tetrabromobisphenol A) from magnetosome.

**Conclusions:**

In this work, removal of most impurities greatly simplified the subsequent purification procedure. The system also facilitated the bioproduction of nanomaterials. The developed platform can substantially simplify industrial protein production and reduce its cost.

## Background

Native and recombinant peptides and proteins are becoming increasingly important as enzymes and non-catalytic functional products (e.g., antibodies, hormones, factors and vaccines) for industrial, nutritional, medical, and agricultural applications [1]. A prokaryotic system is most widely used for protein production at both laboratory and industrial scales. Such a system allows to rapidly produce large quantities of recombinant proteins [2]. Downstream processing refers to the recovery and purification of biosynthetic products and remains a challenge in biotechnology. Protein purification is the most time-consuming and costliest step in protein production [3, 4]. In prokaryotes, purification is more complex because recombinant proteins are mainly produced inside the cells [5]. To purify recombinant proteins in large quantities, it is imperative to ensure effective lysis of cells and separation of target proteins. The most commonly used methods for cell lysis are the enzymatic digestion and physical disruption by sonication or high pressure homogenization. Separation processes are designed to isolate target molecules in the presence of a variety of impurities from a fermentation broth that contains not only many different biomolecules, but also cell debris and salts. The processes typically require numerous steps: filtration, centrifugation, flocculation, sedimentation or crystallization, and chromatographic separation. Those steps require special equipment and often suffer from loss of sample during processing, heat generation, contamination, and high cost. Therefore, downstream processing often reaches to 50–90% of the total production costs in most biotechnological products [6], especially for low yield products.

Magnetic separation utilizes a magnet to attract magnetic substances out of non-magnetic components in a mixture. It enables the fast and direct capture of target molecules in fermentation broths [7]. However, magnetic separation requires high-gradient magnetic fields for target complex separation and hazardous elution buffers such as imidazole to weaken interactions between desired products and magnetic particles [8]. An alternative separation carrier is probably bacterial magnetosome or bacterial magnetic nanoparticle, which has been recently studied for drug delivery [9]. Magnetosomes are special organelles of magnetotactic bacteria such as *Magnetospirillum gryphiswaldense* MSR-1. Magnetosomes are composed of membrane-enveloped magnetite (Fe_3_O_4_) or greigite (Fe_3_S_4_) crystals in diameters ranging from 30 to 120 nm [10]. Magnetosomes have a perfect crystalline core, a quasi-spherical shape and a fair uniform particle size. Their magnetism is generally much stronger than the artificial magnetic nanoparticles of iron oxides [11]. When used as a separation carrier, magnetosomes do not need high-gradient magnetic fields. Magnetosomes are inside bacteria, which can be well used for tailored strategy in downstream processing [12].

The feasibility to fusion express antibodies, fluorophores, enzymes and receptors on magnetosome surfaces (also known as magnetosome surface display) was recently demonstrated and highly attractive for many biomedical and biotechnological applications [13]. Nanobody (Nb) was functionally expressed on the surface of magnetosome via a surface display technique [14]. Nbs are defined as single-domain variable fragments of camelid-derived heavy-chain antibodies. Nbs have arisen as an alternative to conventional antibodies and show great potential in diagnostics and therapy [15]. In 2020, the global Nbs market was worth of U.S. $132 million, and by 2028, it is projected to be worth of U.S. $9,192 million. Nb expression is extensively studied in both prokaryotic and eukaryotic systems, though most of these works fail to reach high production levels so far [16]. However, few reports have been focused on downstream Nb separation and purification up to now.

Tetrabromobisphenol A (TBBPA) is a brominated flame retardant used in 90% of epoxy coated circuit boards [17]. Its potential toxicity to human and bioaccumulation property have brought close attention [18]. An anti-TBBPA Nb immunoassay is a promising method for monitoring TBBPA in the environment [19]. Compared with the classical polyclonal antibody-based immunoassay, the Nb-based immunoassays show excellent sensitivity of TBBPA detection [20].

**Figure 1:**
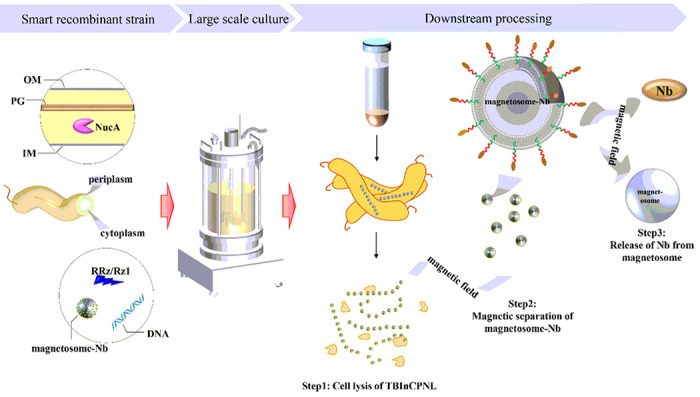
A GEMP for downstream extraction and purification of Nbs. “OM”: out membrane, “PG”: peptidoglycan, “IM”: in membrane.

This study aimed to purify recombinant protein in a simple and economical way, and a novel genetically encoded magnetic platform (GEMP) (Fig. 1), which was capable of simultaneous biosynthesis of proteins of interest (e.g. anti-TBBPA Nb) and automatic separation upon their expression by engineered bacteria, was constructed and evaluated. We chose gram-negative bacteria MSR-1 with magnetosomes as a prokaryotic expression system for subsequent construction to ensure production and automatic separation of recombinant proteins. The system contained a truncated phage lambda lysis cassette (SRRz/Rz1), a non-specific nuclease NucA, a protein-splicing element of *Pyrococcus abyssi* DNA polymerase II (*Pab* PolII intein), and a Nb against TBBPA. The separation procedure of the GEMP included three automatic steps (Fig. 1): cell lysis of bacterial host, magnetic separation of magnetosome-Nb complexes, and release of Nbs from the complexes. Anti-TBBPA Nb was used as a model protein. The results showed a promise for a smart platform for extraction and purification of target proteins.

## Results

### Functional components of GEMP

As a component of GEMP, the phage lambda lysis cassette consisted of four genes: *S, R, Rz* and *Rz1*. The *S* gene encodes holin and its inhibitor. The *R* gene encodes the endolysin. *Rz* and *Rz1* are nested genes encoding Rz and Rz1 proteins, respectively, involved in resolving the oligopeptide linkages. Holin is a small membrane protein, which forms μm-scale holes in host cytoplasmic membrane (inner membrane). These holes result in the release of R endolysin, Rz and Rz1 proteins, thus accessing to their substrate (host cell wall), and at last, the disruption of host cell [21]. The surface attached extracellular nuclease (Nuc) of *Staphylococcus aureus* is a secreted enzyme that possesses a long 60-residue Sec signal sequence. The secreted form of Nuc, known as NucB, is processed by most *S. aureus* strains to a shorter form called NucA [22]. The fusion of NucB and the signal sequence of *E. colis* major outer membrane protein (OmpA) resulted in accumulation of NucA in the periplasm of *E. coli* [23]. NucA can hydrolyze DNA and RNA, whether double or single strand, into oligo- and mononucleotides. NucA was here responsible for reducing extract viscosity by completely digesting genomic DNA [24].

To avoid cell lysis during cultivation, the elements mentioned above need to be expressed at different subcellular locations. The truncated phage lambda lysis cassette and the NucA were expressed in cytoplasm and periplasm, respectively, while the intein and Nb were expressed on the surface of magnetosome (Fig. S1). These elements were cloned into different plasmid vectors, introduced into *E.coli* S17-1 via transformation, followed by function verification of the first two elements. All of vectors was then transferred into MSR-1 via bacterial biparental conjugation.

#### Transfer of nucB and RRz/Rz1 into E. coli S17-1

**Figure 2:**
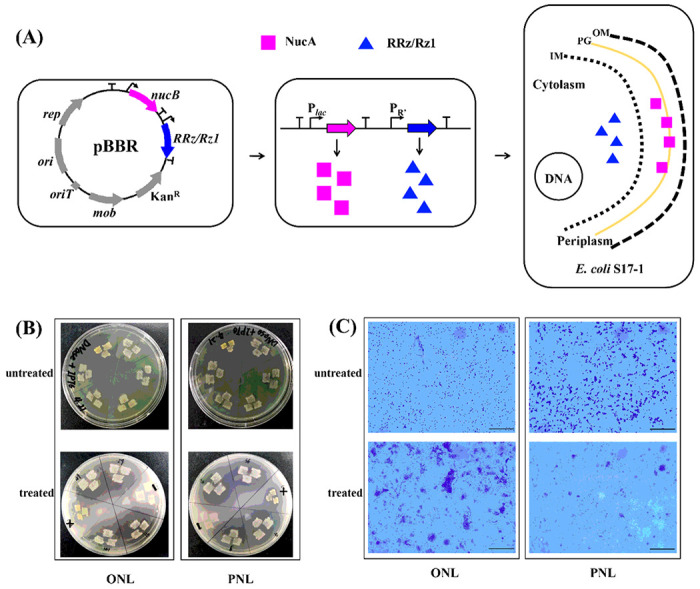
Transfer of *nucB* and *RRz/Rz1* into *E. coli* S17-1. **(A)** Construction of recombinant strains: “OM”: out membrane, “PG”: peptidoglycan, “IM”: in membrane. **(B)** Recombinant strains were cultured on IPTG/DNase agar plates: untreated (Upper), treated with HCI (Lower). **(C)** Lysis of recombinant strains: untreated (Upper); induced with CHCI_3_ (Lower). Scale bar, 50 μm.

In the present work, only *R, Rz* and *Rz1* in the phage lysis cassette were employed and cloned into a plasmid vector pBBR1 MCS-2 to construct recombinant strains. The *S* gene was omitted to avoid host lysis during cultivation. The native promoter P_R_, from λ phage was cloned and employed for the expression of *RRz/Rz1* genes. Besides, the DNA fragment encoding OmpA signal peptide was fused with *nucB*, and then cloned into the plasmid vector pBBR1 MCS-2, along with the *RRz/Rz1*. The *lac* promoter from pBBR1 MCS-2 was employed for the expression of NucA. The resulting plasmid was referred to as pBBRONL (Fig. 2A). Another recombinant plasmid pBBRPNL was also constructed, which was the same as pBBRONL, except that the signal peptide sequence was from a *phaZ1* gene instead of *ompA* (Fig. 2A). The PhaZ1 here was an “intracellular” poly(3-hydroxybutyrate) (PHB) depolymerase of *Rhodospirillum rubrum*, which is a periplasm-located protein with specificity for native PHB and with structural similarity to extracellular PHB depolymerases [25]. *R. rubrum* is closely related to *Magnetospirillum* spp., of which 90% of the 16S rRNA sequence is identical to that of wild type (WT) MSR-1 [26]. The plasmids pBBRONL and pBBRPNL were then transferred into S17-1 and the resulting recombinant strains were referred to as ONL and PNL, respectively (Fig. 2A).

The target genes *nucB and RRz/Rz1* in recombinants were identified by colony polymerase chain reaction (PCR) and sequencing. On isopropyl β-D-1-thiogalactopyranoside (IPTG)/DNase agar plates, transparent zones surrounding the colonies of ONL and PNL were observed, while those of controls (S17-1) did not occur (Fig. 2B). NucA was presumably transported across cytoplasmic membrane into periplasmic space under the direct of signal peptide from OmpA or PhaZ1. To confirm this, two recombinant strains and their host strain (S17-1) were cultivated in shaking flasks. Their cell pellets were harvested by centrifugation and resuspended into 3 mL of Tris-HCl (20 mM, pH 8.0), followed by the addition of CHCl_3_ (20 μL). The majority of ONL and PNL cells was disrupted within 0.5 h, while no apparent changes were observed from S17-1 cells (Fig. 2C), indicating the successful expression of RRz/Rz1 in the cytoplasm of two recombinant strains. Compared to the viscosity of the homogenate from modified S17-1 strains which expressed RRz/Rz1 only, the viscosity from disrupted S17-1 expressing both NucA and RRz/Rz1 was lower (Fig. S2).

#### Construction of an engineered MSR-1 harboring nucB, RRz/Rz1 and Nb

**Figure 3:**
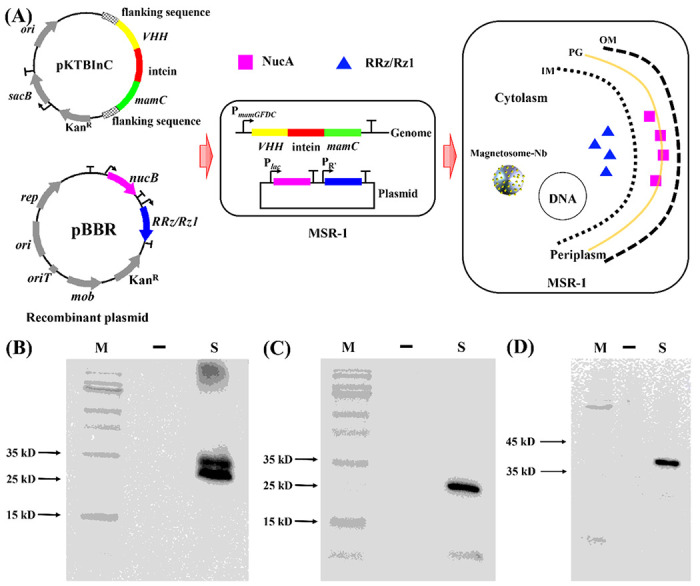
Construction and identification of smart recombinant MSR-1. **(A)** Construction of smart engineering bacteria: “OM”: out membrane, “PG”: peptidoglycan, “IM”: in membrane. RRzRz1 (**B**), NucA (**C**), and Nbs-Intein-MamC (**D**) were detected by western-blot. “M”: marker, “ ”: WT, “S”: TBInCPNL.

*RRz/Rz1* and *nucA* were successfully expressed in *E. coli* S17-1, and their protein products exhibited specific activities in the process of cell self-lysis and nucleic acid hydrolysis. We assumed that *RRz/Rz1* and *nucA* could be expressed in other Gram-negative bacteria including magnetotactic bacteria. Herein, a recombinant MSR-1 was developed for automatic downstream processing. The plasmid pBBRPNL with the *RRz/Rz1* and recombinant *nucB* was employed and transferred into MSR-1. The signal peptide sequence of the recombinant *nucB* gene in this plasmid was from *phaZ1* gene of *R. rubrum*, which was closely related to MSR-1. A fusion gene of the anti-TBBPA Nb, an intein, and MamC was also introduced into MSR-1 via another plasmid vector. The intein gene was cloned from DNA polymerase II of *Pyrococcus abyssi*, abbreviated as the *Pab* PolII intein [27]. MamC was the most abundant protein on the magnetosome surface, which helped to express the anti-TBBPA Nb and the *Pab* PolII intein and anchor them on the surface of magnetosomes [28]. Different from the plasmid pBBRPNL, a suicide plasmid vector pK18mobSacB was employed for cloning the fusion gene of Nb and intein, resulting a recombinant plasmid pKTBInC. After being transferred into MSR-1, the anti-TBBPA Nb and *Pab* PolII intein genes were integrated into the host chromosome. The recombinant MSR-1 strain was referred to as TBInCPNL (Fig. 3A). An additional control strain was also constructed and referred to as TBInC, which contained the fusion gene of anti-TBBPA Nb and *Pab* PolII intein in its chromosome but did not harbor the plasmid pBBRPNL (i.e., without the *RRz/Rz1* and *nucB* genes in this strain).

The western-blot analysis showed that the proteins RRz/Rz1, NucA and Nbs-Intein-MamC extracted from TBInCPNL migrated as expected (Fig. 3B–D), illustrating successful expression of each exogenous gene in respective compartments of the recombinant strain.

### Culture Of Tbincpnl

#### The growth and magnetic response of TBInCPNL in shaking culture

**Figure 4:**
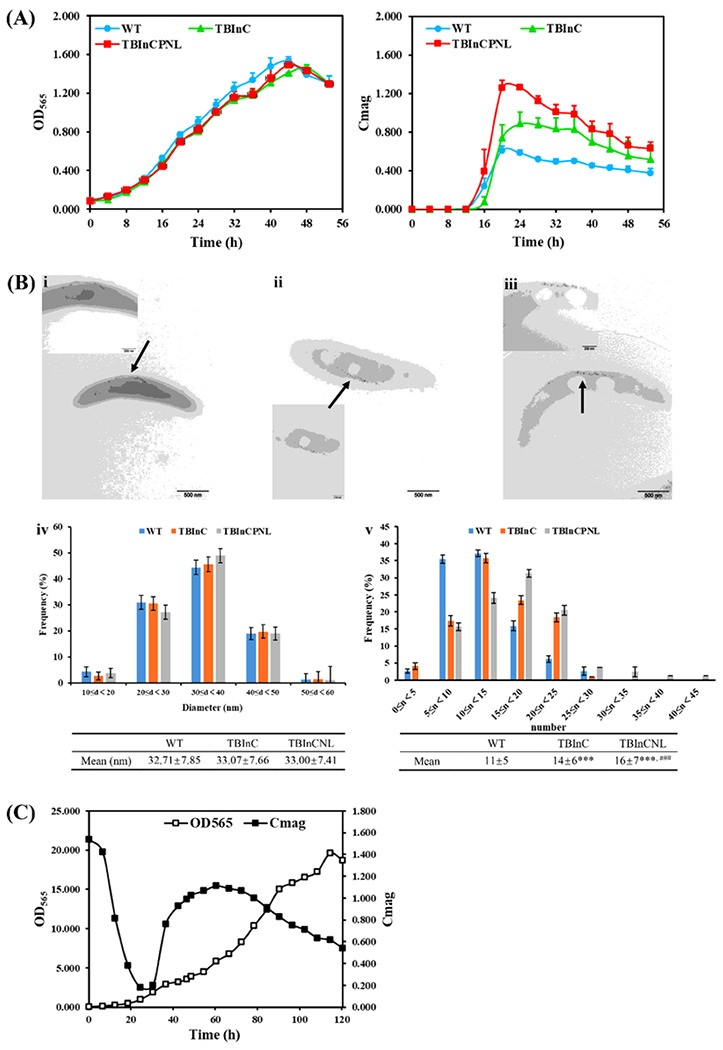
Impacts of exogenous genes on the growth of TBInCPNL and the production of magnetosomes. (**A**) The growth curve (left) and magnetic response curve (right) of strains cultured in a shake flask. (**B**) Strains observed by TEM: (i) WT, (ii) TBInC, (iii) TBInCPNL; and magnetosomes analyzed by ImageJ: (iv) The diameter of magnetosomes, (v) The numbers of magnetosomes in different cells; *** *P* < 0.005 vs. WT, ^###^ *P* < 0.005 vs. TBInC. (C) The growth (OD_565_) and magnetic response (Cmag values) curves of TBInCPNL cultured in a 7.5-L fermenter.

To evaluate the impacts of exogenous genes on the growth of recombinant strains, the OD_565_ value and magnetic response (Cmag) of TBInC and TBInCPNL growing in 100 mL of sodium lactate medium (SLM) were detected and compared with those of WT MSR-1 (Fig. 4A). The growth curves of three strains almost overlapped. The maximum OD_565_ values of MSR-1, TBInC and TBInCPNL were 1.53, 1.46 and 1.49, respectively. After 53 h of cultivation, cells were harvested via centrifugation to yield 0.92, 0.96, and 0.90 g (wet weights, ww) of MSR-1, TBInC, and TBInCPNL, respectively. Although the cell yields varied slightly, the strains showed different magnetic responses. Remarkably, the average Cmag values of TBInC and TBInCPNL were approximately 1.5- and 2-fold higher than that of WT MSR-1, respectively.

#### Magnetosome’ characteristics

The morphology of magnetosomes was characterized by transmission electron microscope (TEM). Magnetosomes from different strains all appeared in a chain (Fig. 4B (i–iii)). The size and yield of magnetosomes were determined with the ImageJ (Fig. 4B (iv–v)). The diameters of magnetosomes were mostly distributed in a range of 20–50 nm (Fig. 4B(iv)). No significant differences were observed in the size of magnetosomes biosynthesized by MSR-1, TBInC, and TBInCPNL, with an average diameter of 32.7 ± 7.9, 33.1 ± 7.7, and 33.0 ± 7.4 nm, respectively (Fig. 4B(iv)). However, dramatic differences were observed in the numbers of magnetosomes biosynthesized by single cell (Fig. 4B(v)). The numbers of magnetosomes in a single cell of WT were distributed in a range of 5–15 and those in a single cell of TBInC and TBInCPNL were distributed in a range of 5–25 (Fig. 4B(v)). The average number of magnetosomes biomineralized in a single cell of WT, TBInC, and TBInCPNL was 11 ± 5, 14 ± 6, and 16 ± 7, respectively (Fig. 4B(v)). These results demonstrated that the transfer of these exogenous genes into MSR-1 exhibited little inhibition on the proliferation of cells but promoted the biomineralization of magnetosomes. It is implied a positive correlation between the magnetic responses and the number of magnetosomes from various strains.

##### Suitability of high cell density cultivation.

Subsequently, TBInCPNL was incubated in fed-batch cultivation in a 7.5-L fermenter. Figure 4C showed a typical growth curve including the lag, exponential, stationary, and decline phases. The peak value of OD_565_ was 19.6, appearing at 114 h. The curve of magnetic response from growing TBInCPNL could be divided into two parts: a decreasing curve and a parabolic curve. Typically, MSR-1 biosynthesizes magnetosomes under a low concentration of dissolved oxygen (dO_2_ < 1 %). When TBInCPNL cells were transferred into the fermenter, dO_2_ of the culture medium was enhanced and the biosynthesis of magnetosomes in cells was temporarily inhibited, leading to the initial decrease of Cmag. After around 25 h culture, dO_2_ was gradually driven down to a level suitable for the biomineralization of magnetosomes and thus, Cmag values started to increase. The Cmag value reached to the peak 1.11 and thereafter declined again with the increase of dO_2_, due to the high stirring rate in partial. After 120 h, TBInCPNL was harvested and the yields of cells and magnetosomes were 106.3 g and 6.8 g (ww), respectively. Hence, in spite of exogenous genes, with *nucB* and *RRz/Rz1* in particular, it was not a problem to carry out a high-density culture of TBInCPNL at a large scale.

### Cascade Cell Lysis Of Tbincpnl And Hydrolysis Of Nucleic Acids

**Figure 5:**
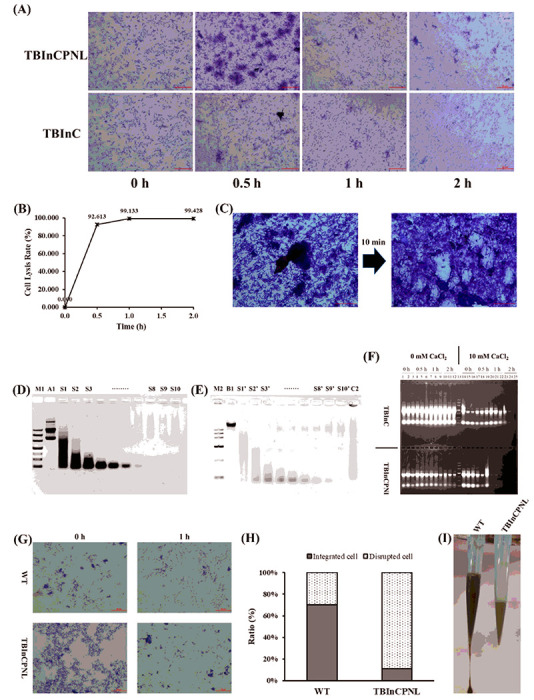
Functions of RRz/Rz1 and NucA expressed in MSR-1.

The function of *RRz/Rz1* and *nucB* had been demonstrated in the recombinant *E. coli* strains (PNL and ONL) above in the section of 3.1. We investigated whether these genes worked in the recombinant MSR-1 strain (TBInCPNL). TBInC, the recombinant strain harboring *Nb* but not *RRz/Rz1* and *nucB*, was employed as a control. Cells of TBInC and TBInCPNL were harvested from a shake-flask culture (100 mL) and resuspended in 3 mL of phosphate buffered saline (PBS: 10 mM, pH 7.4), followed by the addition of 20 μL of CHCl_3_. After 2 h incubation, the majority of TBInCPNL cells were broken, whereas TBInC cells changed slightly (Fig. 5A). The lysis rate of TBInCPNL cells was over 90% within 0.5 h and approximately 99% within 1 h, as determined with a blood counting chamber (Fig. 5B). Another method also showed that RRz/Rz1 worked well in TBInCPNL. When the frozen TBInCPNL cells were transferred from liquid nitrogen to room temperature, they had almost been completely lysed within 10 min (Fig. 5C). These results indicated that RRz/Rz1 was functional in TBInCPNL, and cascade cell lysis would occur at the suitable condition (e.g., CHCl_3_ or liquid nitrogen).

Extracts from the periplasmic space of TBInCPNL by osmotic shock were able to hydrolyze the plasmid pBBRPNL and genomic DNA of MSR-1 at 37°C (Fig. 5D and E), indicating the expression of the functional NucA. After the addition of lysozyme into the cell suspension of TBInC and TBInCPNL (details in Materials and Methods), the mixtures were incubated at room temperature for 2 h. In the absence of Ca^2+^, nucleic acids in homogenates of both TBInC and TBInCPNL could be detected within 2 h. While in the presence of Ca^2+^, nucleic acids in the homogenate of TBInCPNL were hardly detectable after incubation for 1 h, but detectable in the homogenate of TBInC within 2 h (Fig. 5F). These results illustrated that NucA was expressed in the periplasmic space of TBInCPNL and showed a strong non-specific hydrolysis capability for nucleic acids in the presence of Ca^2+^ at room temperature.

The activities of NucA and RRz/Rz1 were further evaluated in TBInCPNL cells cultivated in a 7.5-L fermenter. Here, WT MSR-1 was used as a control. Cells were harvested at the later stage of exponential phase of the growth curve and then treated with CHCl_3_. One hour after the addition of CHCl_3_, over 75% WT cells were intact, while approximately 90% TBInCPNL cells were disrupted (Fig. 5G and H). In a gravity flow experiment [29], the homogenate of disrupted TBInCPNL cells had a lower viscosity than that of WT cells (Fig. 5I), indicating that the cascade cell lysis and hydrolysis of nucleic acids occurred in TBInCPNL from the large-scale culture.

### Extraction And Isolation Of Nbs

**Figure 6:**
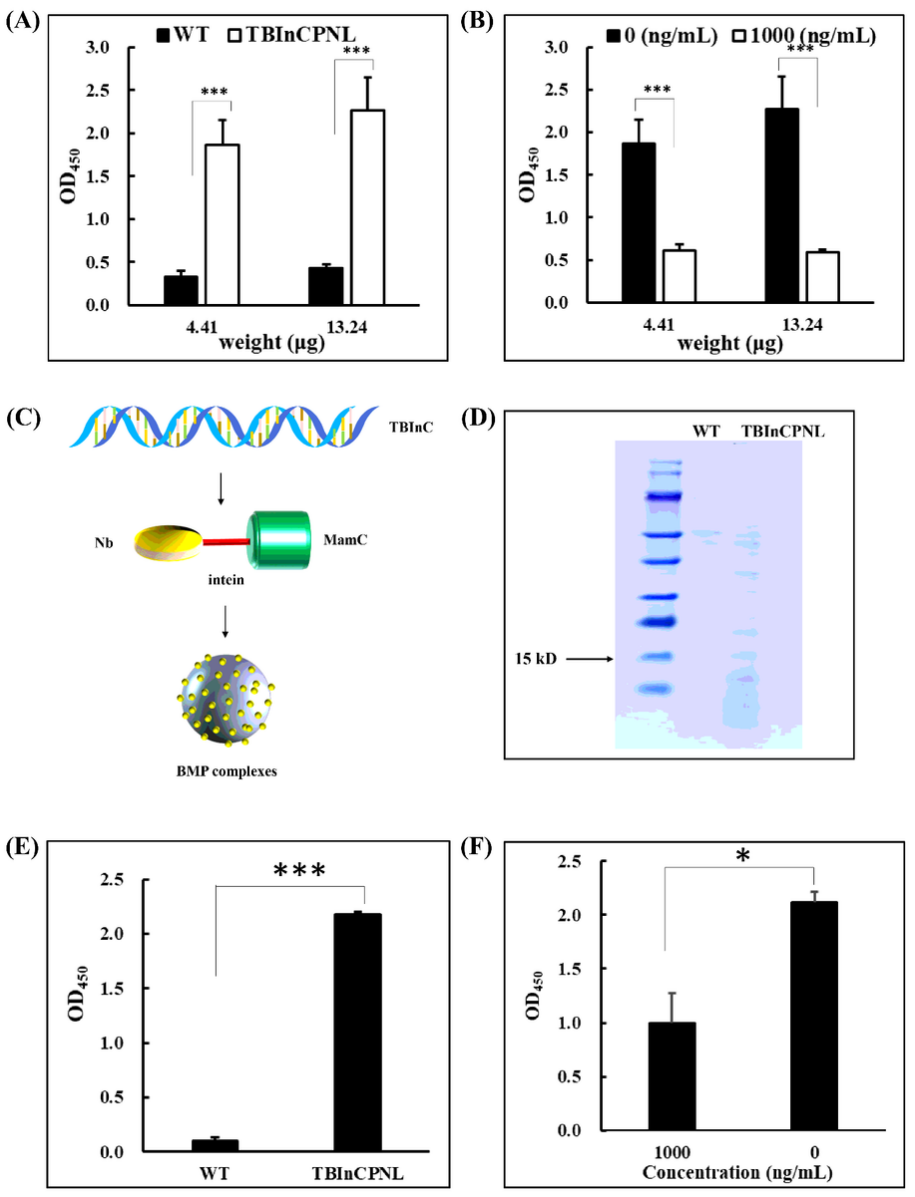
Identification of anti-TBBPA Nbs expressed in the recombinant strain TBInCPNL. (**A**)Binding activity of magnetosome-Nb complexes to T5-HRP by a non-competitive ELISA. (**B**) Binding activity of magnetosome-Nb complexes to TBBPA by a competitive ELISA. (**C**) Formation of the complex MamC-Intein-Nbs. (**D**) The Nb detected on an SDS-PAGE. (**E**) Binding activity of Nbs in supernatant to T5-HRP by a non-competitive ELISA. (**F**)Binding activity of Nbs in supernatant to TBBPA by a competitive ELISA.

In general, the cells should be harvested before a drastic drop of Cmag values to ensure a high yield of magnetosomes. Herein, when the Cmag values of TBInCPNL dropped to approximately 1.0 from the peak, cells were harvested even though they were still in the exponential phase of growth. According to the curves of magnetic response and cell growth (Fig. 4C), a 5-L cultivation of TBInCPNL was harvested at 72 h (OD_565_ = 8.28, Cmag = 1.07). Cells were separated from the culture medium by centrifugation. The medium supernatant was then concentrated to approximately 25 mL, containing proteins at a concentration of 9.05 mg mL^− 1^. The proteins in the supernatant showed no binding activity to TBBPA or its hapten T5 conjugated with horseradish peroxidase (HRP) (T5-HRP) by enzyme-linked immunosorbent assays (ELISAs), suggesting that Nbs were hardly secreted into medium. Afterwards, the cascade-amplified lysis of cells suspended in PBS (with Ca^2+^) was carried out using CHCl_3_. Magnetosomes were separated from cell broth under a magnetic field and cleaned up by washing with PBS (pH 7.4). The resultant magnetosomes exhibited a strong binding activity to T5-HRP by a non-competitive ELISA (Fig. 6A), and to TBBPA by a competitive ELISA (Fig. 6B). The results indicated the attachment of Nbs to magnetosomes. The cell broth debris was removed via centrifugation. The binding activities of proteins in the supernatant and precipitant to antigens (TBBPA or T5-HRP) were evaluated. No obvious binding activities were observed. Then the ratios of Nbs in supernatant (soluble proteins) and precipitant (insoluble proteins) with whole cell were further analyzed at two different stages: i) In exponential phase (OD_565_ = 8.28, Cmag = 1.07), only ~ 2% Nbs were detected in the supernatant and ~ 8% Nbs were detected in the precipitant (Fig. S3A); ii) In decline phase (OD_565_ = 18.72, Cmag = 0.54), only ~ 1% Nbs were detected in the supernatant and ~ 14% Nbs were detected in the precipitant (Fig. S3B). These results supported that the majority of Nbs was attached to magnetosomes.

In the present study, the Nb was immobilized on the surface of magnetosomes, via a *Pab* Pol intein as a bridge of Nb and MamC, to form a fusion protein on magnetosome, MamC-Intein-Nb (Fig. 6C). *Pab* PolII intein could promote protein splicing *in vitro* at high temperature [27]. Therefore, the temperature controlled self-splicing of intein would be accompanied with the separation of Nbs from magnetosomes. Under the optimized cleaving conditions: 0.1 g magnetosome complexes (MamC-Intein-Nb) were suspended in 300 μL of PBS (pH 6.0) containing 200 mM dithiothreitol (DTT) and incubated at 50°C for 30 min. Nbs were detected in PBS by sodium dodecyl sulphate-polyacrylamide gel electrophoresis (SDS-PAGE) analysis (Fig. 6D) and showed binding activities to T5-HRP and TBBPA (Fig. 6E and F). Magnetosome complexes showed a slight binding capability to TBBPA after the splicing treatment (data not shown), indicating that Nbs were split from the complexes. The yield of Nbs released from magnetosome complexes was approximately 0.60 μg mg^− 1^.

## Discussion

Advances of molecular biology have improved cloning and culture methods, which has diversified applications of recombinant proteins, ranging from enzymes used in laundry detergents to antibodies employed in cancer therapy [1]. Extensive studies have been carried out to find suitable production systems for high-level expression of recombinant proteins [30, 31]. However, downstream processing often contributes more to production cost than upstream and fermentation process [8]. Most efforts were previously focused on extracellular expression or production to reduce the downstream processing cost, but in general, the yields of extracellular protein were much lower than those produced intracellularly [32]. The downstream processing of intracellular products needs multiple steps, including host cell harvest, cell disruption, and target protein isolation and purification. Though there are several reports on controllable cell lysis [33], no complete genetic engineering platform for downstream processing was available.

The methods for the productions of recombinant proteins are quite diverse. The recombinant proteins are often produced in high yields with maturity technology, while the production capacities of newly constructed recombinant proteins are usually low [32]. Developing a system for the isolation of low-level product, in a sense, is a more difficult task, because it must remove much larger proportion of impurities. In this work, we developed a complete set of genetic engineering downstream processing platform for low expression level proteins, from cell harvest to target protein isolation. Since the magnetotactic bacteria strain MSR-1 was employed as the host strain, the cells with target proteins could be harvested by magnetic separation, instead of traditional centrifugations. Magnetic separation makes the collection of host cells much easier for continuous pipeline transportation, which is a progressive and economically advantageous mode of industrial transportation [34]. Cell disruption is also a key downstream processing step. This is a costly step for industrial production, in which high pressure homogenization was often employed. Autolysis or controllable lysis of host cell is the most convenient way to release intracellular products [35]. However, after cell lysis, the release of long chain nucleic acids led to a very high viscosity, which makes difficult for the next downstream processing steps [36]. We here developed a surface expressed nuclease to degrade nucleic acids and decreased the viscosity, which would simplify the rest of downstream processing steps (Fig. 1). The chromatographic procedure has been used in the isolation and purification of most protein products, as it has the advantages of having mild elution conditions and strong and specific binding. However, in the industrial scale, these methods generally need large equipment, and complicated and laborious procedures for chromatographic matrix cleaning and regeneration [37]. The anti-TBBPA Nb was expressed on the surface of magnetosomes in this work. Nbs could be retrieved by magnetic separation from cell debris and undesired molecules in the host crude extract. The Nb was then abscised via intein on the engineered magnetosomes, and thus separated the protein product and magnetosome (Fig. 1). Although the product Nbs need further purification in this work, most impurities were removed (Fig. 6D), which greatly simplifies subsequent purification procedures. This system could also be employed to express insoluble proteins or membrane proteins. In traditional systems, these proteins usually accumulated in cytoplasm as inclusion bodies. While, in this system, the recombinant protein was expressed on the magnetosome surface instead of forming inclusion bodies. Besides, the system also facilitates bio-nano engineering and the bioproduction of functional nanomaterials, such as metal, alloy, or metallic oxide. These rigid materials, in traditional procedures, often cause serious wear of high-pressure homogenizer during host cell disruption [38]. In contrast, the damage from the homogenization could be completely avoided in the system described here.

The host strain, MSR-1, also has the potential for high cell density cultivation. Traditionally, cultures with high microbial cell density have a high metabolic oxygen demand. In these cultures, the oxygen transfer rate of the bioreactor determines the maximum biomass concentration. Unfortunately, the solubility of oxygen decreases with increasing cell densities due to higher viscosity of the cultivation. Therefore, high speed agitation and a large amount of pure oxygen are required at the latter part of cultivation, which is an energy-intensive and costly process [39]. MSR-1 is a microaerophilic strain. Compared with prevalent host strains, such as the strains of *E. coli, Bacillus subtilis*, or *Saccharomyces cerevisiae*, MSR-1 demands much less oxygen during cultivation when reaching the same cell density [40, 41], which made the procedure energy-efficient and economical. It has been reported that the stirring speed was over 750 rpm, when the culture density of *E. coli* reached to an OD_600_ of 20 [39]. While, at similar cell density, only around 400 rpm was required for MSR-1 cultivation. Fortunately, MSR-1 is also a typical gram-negative bacterium like *E. coli*. Most plasmid vectors, genetic elements, and gene expression systems of *E. coli* also work in MSR-1. We thus believe a good possibility to construct a microaerobic gene expression system of MSR-1 in the near future, which will make a great progress of recombinant protein production [9]. Nonetheless, much effort is required to develop a gene expressing system in a microaerophilic bacterium, especially to set up easier cultivation methods, well-developed genetic manipulation system. Besides, magnetosome numbers in each cell and the percentage of MamC in the membrane proteins need to be substantially increased to achieve high protein productions.

## Conclusion

We constructed a new complete genetic engineering downstream processing platform for low expression level of proteins in the magnetotactic bacterium MSR-1. This smart magnetosome-based platform was efficiently controlled by three functional components including RRz/Rz1, NucA, and magnetosome complex (MamC-Intein-Nbs), which were amenable to the lysis of host cell, the reducing viscosity of homogenate, and the separation and purification of Nbs, respectively. Using this platform, most impurities were removed from the medium and the subsequent purification procedure of Nbs was greatly simplified. Such a platform is an innovative protein purification platform to advance and overcome current bioprocessing challenges.

## Materials And Methods

### Bacterial strains and culture conditions

The bacterial strains and plasmids used in this study are listed in Table S1. *Escherichia coli* strains were cultured in Luria broth (LB) or NDase Agar plates for detecting NucA at 37°C. MSR-1 cells were cultured in SLM with 20 μM ferric citrate or sodium glutamate medium (SGM) at 30°C (substitute 4 g SGM for NH_4_Cl and yeast extract of SLM) [42]. MSR-1 cells were cultured in 100-mL serum bottles filled with 50 mL of medium or 250-mL serum bottles filled with 100 mL of medium. In addition, MSR-1 cells were cultured in a 7.5-L fermenter with Fed-batch culture described as previous [43]. Antibiotics used were as follows: for *E. coli*, ampicillin at 100 μg mL^− 1^ and kanamycin (Km) at 50 μg mL^− 1^; for MSR-1, Km at 5 μg mL^− 1^ and nalidixic acid at 5 μg mL^− 1^. The growth (OD_565_) and Cmag of MSR-1 were measured as described previously [44].

### Plasmid And Strain Construction

All plasmids used in this study are listed in Table S1. All cloning was performed in *E. coli* using restriction enzyme ligation (TaKaRa, Japan). For PCR amplification, 2 × Phanta Max Master Mix (Vazyme, China) were used with the primers listed in Table S2.

*nucB* was amplified from *S. aureus* NCTC 8325 genomic DNAthat the signal peptide from *E. coli* OmpA or *R. rubrum* PhaZ1 was expressed at N-terminal. *RRz/Rz1* expressed using the native promoter P_R_, was amplified from λ phage genomic DNA. HA and Flag tags were fusion expressed at C-terminal of NucB and RRz/Rz1, respectively. Then *nucB* and *RRz/Rz1* were assembled into a cassette by fusing PCR amplification, named as *ONL* (signal peptide from *E. coli* OmpA) or *PNL* (signal peptide from *R. rubrum* PhaZ1). *ONL* and *PNL* were inserted into the plasmid pBBR1MCS-2 digested with *BamH* I and *Xba* I using T4 DNA ligase to generate recombination plasmid pBBRONL and pBBRPNL, respectively. Finally, pBBRONL and pBBRPNL were transferred into *E. coli* S17-1, named as ONL and PNL, respectively.

*mamC* and its upstream and downstream homology regions were amplified from MSR-1 genomic DNA. Anti-TBBPA Nb with 6×his tag at its C-terminal was amplified from a plasmid (pecan 45) containing the Nb-alkaline phosphatase (AP) fragment [45]. The fusion gene consisted of *Nb* and *mamC* was cloned by fusing PCR amplification with primers TB-F (*BamH*I) and C-R (*Xba* I), named as *TBC. uTBCd*, a cassette of *mamCs* upstream region, *TBC*, and *mamCs* downstream region, was assembled with fusing PCR amplification. The gene sequence coding *Pab* Pol intein, synthesized with Myc tag expressed at C-terminal by Tsingke Biotechnology Co., Ltd., was inserted into *uTBCd* to generate *uTBInCd* by PCR amplification. Finally, *uTBInCd* was subcloned into pK18mobSacB with *EcoR* I and *Xba* I to create recombination suicide plasmid pKTBInC, which was transferred into *E. coli* S17-1 and then WT MSR-1 by biparental conjugation to obtain TBInC. The smart engineering strain was referred to as TBInCPNL generated from TBInC and PNL by biparental conjugation.

### Target Protein Expression In Different Compartments (Periplasmic Proteins, Cytoplasmic Proteins, And Magnetosome Membrane Proteins)

Strains were cultured in shake flasks or serum bottles and harvested under these conditions: for *E. coli*, IPTG adding to culture medium reach to 1 mM at OD_600_ 0.4-0.6 and harvesting 3–5 h later, and for MSR-1, IPTG adding to culture medium reach to 0.3 mM at OD_565_ ~ 0.4 and harvesting at OD_565_ ~ 1.0.

Periplasmic proteins were extracted according to the osmotic shock procedure. First, cell pellets were washed two times with deionized water. The cell pellets were then resuspended in a solution containing 200 mM Tris-HCl (pH 8.0), 1 mM ethylene diamine tetraacetic acid (EDTA), and 20% (w/v) sucrose, incubated with a slow shaking at room temperature for about 10 min, and harvested at 8,000*g* for 10 min. After removing supernatant, the cell pellets were resuspended in cold Tris-HCl (200 mM, pH 8.0) again, and incubated on ice (mixing every 2 min) about 10 min. Finally, the precipitate (spheroplasts) and supernatant (periplasmic proteins) were separated by centrifugation (8,000*g*, 4°C, 10 min).

MSR-1’s spheroplasts were broken up to separate cytoplasmic proteins and magnetosome. The spheroplasts (small volume) were resuspended in Tris-HCl (200 mM, pH 8.0) and lysed with ultrasonic (power: 200 W → 100 W → 40 W, every assigned power total working time: 30 min, every working cycle: working 3 s, stop 5 s). Cytoplasmic proteins and magnetosomes were then separated with a magnet. Magnetosomes were thoroughly washed with PBS (10 mM, pH 7.4) to remove cytoplasmic impurities and retrieved by a magnet until protein contents in supernatant varied negligibly. Periplasmic proteins, cytoplasmic proteins, and magnetosome membrane proteins were separated on SDS-PAGE. Target proteins were detected by western blotting with mouse monoclonal antibodies (anti-HA tag, anti-Flag tag, anti-His tag, and anti-Myc tag).

### Cell Lysis

Cells (1−3 g) were resuspended in PBS (or Tris-HCl) with various volumes (0.5, 1, 2, 3, 4, and 5 mL), and incubated with CHCl_3_ (20, 50, 100, 200, 300, and 400 μL) within stipulated time (0, 0.5, 1, 1.5, and 2 h). The cells were observed under a microscope (Nikon, H550S, made in Japan) and recorded with a blood counting chamber.

### Nucleic Acid Hydrolysis

#### Non-specificity:

Total proteins were extracted from periplasmic space of recombinant strains and then incubated with genomic DNA from MSR-1 or plasmids overnight at 37°C. The mixtures were subjected to agarose gel electrophoresis. The concentrations of total proteins were determined with Bradford Protein Assay. The concentrations of genomic DNA or plasmids were determined on a Nanodrop-1000 (Thermo scientific, American).

Hydrolysis capability of nucleic acids in homogenate: Total nucleic acids were extracted from homogenates of broken cell according to the method of phenol-chloroform-isopentanol (v: v: v = 25:24:1). Strains were cultured in shake flasks (or serum bottles) with 100 mL of medium, harvested at 1.0 of OD_565_, and lysed by lysozyme (20 mg mL^− 1^ lysozyme, 20 mM Tris, 1.2% TritonX-100, pH 8.0) with or without 10 mM CaCl_2_. The lysate was used to detect the action of NucA expressed in engineering strains. The lysate was transferred into a new Eppendorf tube at different time (0, 0.5, 1, and 2 h) and the collection volume was at 200 μM each time. Phenol-chloroform-isopentanol was added with an equal volume (200 μL), vibrated drastically for 10 s, and centrifuged at 12,000*g* for 5 min. The supernatant was transferred into another new Eppendorf tube again. Sodium acetate (3 M, pH 5.2) that was 1/10 volume supernatant and ice-cold anhydrous alcohol that was 2-2.5 volume supernatant were successively added, mixed, maintained at least 5 min on ice, and centrifugated at 12,000*g* for about 10 min. The precipitates were washed twice with 70% alcohol (1 mL) after supernatant moved. Nucleic acids were dissolved in ddH_2_O and detected with agarose gel electrophoresis.

#### Viscosity:

The viscosity of homogenate from cells incubated with CHCI_3_ or lysis buffer (200 mM NaOH, 1 % SDS (w/v)) were tested by gravity flow experiments [29].

### Transmission Electron Microscopy

Cells were collected at ~ 1.0 of OD_565_, placed on copper grid, washed thrice with ddH_2_O, and observed under a JEM-1230 TEM (JEOL, Tokyo, Japan). Numbers and diameters of magnetosomes were analyzed statistically with ImageJ (National Institutes of Health; Bethesda, MD, USA), a Java-based image-processing program.

### Conjugation Of Tbbpa Derivative To Protein

The hapten (T5) of TBBPA (Fig. S4) was available from our previous study [46] and coupled to HRP or bovine serum albumin (BSA) according to the method described previously [47]. The concentrations of T5-HRP or T5-BSA were determined with BCA protein assay.

### Purification And Quantitation Of Anti-tbbpa Nbs

Strains were cultured in a 7.5-L fermenter according to the fed-batch culture described previously [43]. Magnetosome-Nb complexes were harvested from the cell homogenates (whole cell) under a magnetic field. The rest of homogenates was collected (1 mL) and separated by centrifugation at 4°C with 12,000*g* to generated supernatant (cytoplasm) and precipitate (cell debris). The precipitate was resuspended in 1 mL PBS (pH 7.4). Proteins from whole cell, supernatant and precipitate were separated on SDS-PAGE, detected with western blotting based on His tag at C-terminal of anti-TBBPA Nbs, and analyzed with ImageJ. The ratios of anti-TBBPA Nbs in different components with whole cell proteins were calculated.

Magnetosome-Nb complexes were washed with PBS (10 mM, pH 7.4) and detected by ELISAs (14,19). The total proteins of magnetosome-Nb complexes or magnetosome, extracted with a 2.5 mM Tris-HCI (pH 6.8) solution containing 0.1% SDS and 500 mM DTT, were analyzed by Bradford Protein Assay. Anti-TBBPA Nbs were released from magnetosomes with the N-terminal cleavage of *Pab* Pol intein. Magnetosome-Nb complexes were resuspended in PBS with or without reductant and reacted at various temperature. The concentration and function of anti-TBBPA Nbs released from magnetosome complexes were analyzed with Bradford Protein Assay and ELISA, respectively. The reaction conditions of *Pab* Pol intein were optimized: temperature (4,30,40, 50, and 60°C), reductant DTT (0,10,20, 50,100,150, and 200 mM), time (0, 0.5,1,1.5,2,3, and 4 h), pH (5, 6, 7.4, 8, and 9), and quantities of magnetosome-Nb complexes (9-151 mg).

## Data Availability

Data sharing not applicable to this article as no data-sets were generated. Data analysis in the current study was performed using publicly available datasets.

## Abbreviations

GEMP: Genetically encoded magnetic platform
Nb: Nanobody
TBBPA: Tetrabromobisphenol A
OM: Out membrane
PG: Peptidoglycan
IM: In membrane
OmpA: Outer membrane protein
PHB: Poly(3-hydroxybutyrate)
WT: Wild type
PCR: Polymerase chain reaction
IPTG: Isopropyl β-D-l-thiogalactopyranoside
SLM: Sodium lactate medium
TEM: Transmission electron microscope
Ww: Wet weights
HRP: Horseradish peroxidase
ELISAs: Enzyme-linked immunosorbent assays
SDS-PAGE: Sodium dodecyl sulphate-polyacrylamide gel electrophoresis
LB: Luria broth
SGM: Sodium glutamate medium
Km: Kanamycin
AP: Alkaline phosphatase
EDTA: Ethylene diamine tetraacetic acid
BSA: Bovine serum albumin
DTT: Dithiothreitol
Nuc: Nuclease
PBS: Phosphate buffered saline
